# Pregnancy-related pelvic girdle pain affects balance in the second and third trimesters of pregnancy

**DOI:** 10.1371/journal.pone.0287221

**Published:** 2024-03-08

**Authors:** Ivana Hrvatin, Darja Rugelj, Darija Šćepanović

**Affiliations:** 1 Faculty of Health Sciences, University of Ljubljana, Ljubljana, Slovenia; 2 Gynaecological Clinic, University Medical Centre Ljubljana, Ljubljana, Slovenia; West Virginia University, UNITED STATES

## Abstract

**Introduction:**

During pregnancy, many changes in the musculoskeletal system and pregnancy-related disorders affect posture and postural stability. Pregnancy-related pelvic girdle pain (PPGP) is a common disorder in pregnancy; the cause remains unknown. The purpose of the present study was to determine if PPGP affects static postural stability and its relation to the stage of pregnancy.

**Methods:**

Sixty-three pregnant women between the ages of 18 and 45 and between the 12^th^ and 38^th^ weeks of gestation were included in the study. They were divided into four groups according on the trimester and the presence of PPGP. Static balance was assessed using a force plate on firm and compliant surfaces with eyes open and closed.

**Results:**

Pregnant women with PPGP had significantly (p < 0.05) greater centre-of-pressure velocity and sway area compared to pregnant women without PPGP, especially in the third trimester of pregnancy. In the second trimester, only two significant differences in COP parameters were observed between pregnant women with and without PPGP. Pregnant women in the third trimester of pregnancy had significantly (p < 0.05) greater centre-of-pressure velocity and larger postural sway area compared to pregnant women in the second trimester of pregnancy, regardless of PPGP.

**Discussion and conclusion:**

Pregnant women with PPGP had poorer static stability when compared to pregnant women without pain, especially in the third trimester of pregnancy. The cause could be found in the poorer ability to stabilise the trunk and pelvis, poorer proprioception, and issues with automatic movement patterns.

## Introduction

Pregnancy is associated with various hormonal, anatomical, and physiological changes. Significant physiological changes occur in the cardiovascular, respiratory, haematological, renal, gastrointestinal, and endocrine systems to enable the development of the foetus [1]. With the advancement of pregnancy, many changes occur in the musculoskeletal system, causing postural changes and increasing the risk of falling [2]. Between 25% and 27% of pregnant women experience a fall during pregnancy [3, 4], a rate similar to women aged between 70 and 80 years [3]. The majority of falls occur between the 5^th^ and 7^th^ months of pregnancy, while the risk of falling increases during pregnancy [5]. Falls are the primary reason for more than half of maternal injuries during pregnancy [6, 7]. In a systematic review [8], 13 intrinsic and 11 extrinsic risk factors for accidental falls during pregnancy were identified. The identified intrinsic factors include age less than 30, body height more than 160 cm, advanced pregnancy, unintended pregnancy, multiparity, hyperemesis gravidarum, low back pain, gestational diabetes, increase in abdominal circumference, lower ankle stiffness, and joint laxity [8].

Pregnancy significantly affects static stability in the third trimester of pregnancy, demonstrated by the greater centre of pressure (CoP) path length in pregnant women compared to non-pregnant women [9–11]. Some studies have also demonstrated significant differences in the second trimester [9, 11, 12], while no differences have been observed in the first trimester of pregnancy [9, 11, 12]. Pregnant women rely significantly on visual cues to maintain static stability [1, 9, 12–14] due to the impaired perceptions of the proprioceptive system [13].

Due to changes in the musculoskeletal system and posture during pregnancy, some adverse effects, such as pelvic girdle and low back pain, may occur [15]. Pelvic girdle pain is located between the posterior iliac crest and the gluteal fold, particularly in the vicinity of the sacroiliac joints, which can radiate from the posterior thigh to the knees. It can be associated with pubic symphysis pain [16]. The cause of pregnancy-related pelvic girdle pain (PPGP) remains unknown, although it is most likely caused by centre-of-mass (COM) changes, increased load due to weight gain, and decreased pelvic stability due to hormonal changes [17]. The prevalence of PPGP is 22.5% [18], 10% of pregnant women have mild symptoms, while 2.5% have severe symptoms [19]. PPGP usually occurs between the 17^th^ and 19^th^ weeks of gestation with the highest intensity between the 24^th^ and 36^th^ weeks of gestation [20]. Risk factors for PPGP include heavy workload, previous history of low back pain, a history of pelvic girdle or low back pain in previous pregnancies and injuries to the pelvis [16, 18, 21]. PPGP is often considered a normal part of pregnancy due to health workers’ lack of knowledge about possible treatment options [22]. Treatment options include physiotherapy, pharmacological treatment, the use of pelvic belts, and other complementary treatments such as acupuncture and massage [16, 23].

To our knowledge no previous study investigated the effect of PPGP on postural stability during pregnancy. As PPGP could be caused by COM changes and decreased pelvic stability [17], it could further affect postural stability. This study aimed to determine if pregnant women with PPGP have poorer postural stability in the second and third trimesters of pregnancy compared to pregnant women in the second and third trimesters of pregnancy without PPGP and to determine if women in the third trimester of pregnancy demonstrate decreased postural stability compared to that of the women in the second trimester of pregnancy.

## Methods

The study was approved by the Slovenian Medical Ethics Committee (No. 0120-53/2020/7). Prior to any measurement, participants read information about the test protocol and received additional verbal explanations, when required. All participants provided their written informed consent.

### Participants

Pregnant women with singleton pregnancies aged between 18 and 45 years and between the 12^th^ and 38^th^ weeks of gestation were eligible to participate in the study. Exclusion criteria were high-risk pregnancy confirmed by a physician, other musculoskeletal or neurological disorders that can affect balance, history of low back or pelvic girdle pain prior to the present pregnancy, balance impairments, deformations of the spine, injuries or surgery on lower limbs, spine or pelvis. All pregnant women who were unable to walk or stand were also excluded. PPGP in the current pregnancy was not an exclusion criteria.

All pregnant women in the second (between the 12^th^ and the 26^th^ gestational week) and third trimester (between the 27^th^ and 38^th^ gestational week) of pregnancy who attended their regular check-up with their gynaecologist were invited to participate to the study. Pregnant women were interviewed and screened for eligibility to participate to the study. Women were interviewed regarding the current gestational week, number of births and pregnancies prior to the current pregnancy, if they lifted object heavier than 5 kg during the current pregnancy often, sometimes or never, falling rate and its causes during the current pregnancy, physical activity before pregnancy and in the last two weeks before the interview, if they have standing, sedentary or mixed work position and sick leave during the current pregnancy. Participants were considered to be physically active if they were physically active at least 2-times per week for at least one hour.

### Group formation

Sixty-three (63) pregnant women were included in the study. The mean age was 30.3 years, the mean body mass was 72,6 kg and the mean body height was 166.9 cm. The women who met the inclusion criteria were assigned into one of the four groups depending of the current gestation week as well as presence of PPGP. Gestation week information was obtained during the initial interview, from the participants. Pregnant women between the 12^th^ and 26^th^ week of gestation were included in the second-trimester group, while those between the 27^th^ and 38^th^ week of gestation were included in the third-trimester group. Thirty pregnant women were in the second trimester of pregnancy; 33 were in the third trimester.

Afterwards all women were tested by a physical therapist to determine the presence of PPGP according to European guidelines for the diagnosis of PPGP that include six tests. The included tests were the active straight leg test, the posterior pelvic provocation test, the FABER test, the modified Trendelenburg test, the long dorsal sacroiliac ligament test and the symphysis pain palpation test [16]. Women that tested positive in at least three of six performed tests and complained of pain in the pelvic girdle region were included in the PPGP group, thus forming the four testing groups: the second trimester PPGP and non-PPGP groups, and the third trimester PPGP and non-PPGP groups. Thirty-two (32) pregnant women had PPGP, 15 in the second trimester and 17 in the third trimester of pregnancy. The intensity of pain was evaluated using the visual analogue scale (VAS). Cases and controls were not matched for BMI, body mass or age. Details of the participants are presented in Table 1.

**Table 1:** Demographic characteristics of participants with and without PPGP across 2nd and 3rd trimesters.

	Second trimester		Third trimester		ANOVA
	NP (N = 15)	PPGP (N = 15)	NP (N = 16)	PPGP (N = 17)	
	Average (SD)	Average (SD)	Average (SD)	Average (SD)	
**Age (years)**	30.1 (5.4)	29.9 (4.7)	30.9 (6.5)	30.3 (5.5)	0.96
**Body height (cm)**	166.5 (5.0)	167.5 (7.1)	166.3 (5.8)	167.1 (3.9)	0.92
**Body mass (kg)**	69.1 (15.6)	68.7 (12.0)	75.1 (11.1)	76.8 (16.1)	0.25
**N pregnancies**	2.5 (1.8)	2.0 (1.2)	1.3 (0.7)	1.8 (0.8)	0.06
**N births**	1.4 (1.7)	0.9 (0.9)	0.2 (0.5)	0.8 (0.7)	0.03*^1^
**Week of gestation**	19.1 (3.6)	19.9 (3.7)	33.6 (3.9)	33.6 (9.3)	0.00*^2^
**Falls N (%)**	1 (6.7%)	5 (33.3%)	2 (12.5%)	5 (29.4%)	0.19
**PA before pregnancy N (%)**	12 (80%)	10 (67%)	9 (56.3%)	10 (58.8%)	0.52
**PA in pregnancy N (%)**	9 (60%)	4 (27%)	6 (37.5%)	3 (17.6%)	0.08
**Workplace**					0.64
**Sitting N (%)**	8 (54%)	7 (47%)	9 (56%)	7 (41%)	
**Standing N (%)**	5 (33%)	3 (20%)	4 (25%)	8 (47%)	
**Both N (%)**	2 (13%)	5 (33%)	3 (19%)	2 (12%)	
**Sick leave N (%)**	2 (13%)	4 (27%)	0 (0%)	4 (23.5%)	0.79
**Lifting**					0.04*^3^
**Never N (%)**	11 (74%)	7 (47%)	10 (62%)	5 (29%)*	
**Sometimes N (%)**	2 (13%)	7 (47%)	4 (25%)	5 (29%)*	
**Often N (%)**	2 (13%)	1 (6%)	2 (13%)	7 (42%)*	

NP–pregnant women without pain; PPGP–pregnant women with pregnancy-related pelvic girdle pain; N–number; PA–physical activity at least two times weekly for at least one hour; SD–standard deviation; *—statistically significant value (p<0.05).

Note: *^1^Using the Fischer LDS Post-Hoc test a significant difference was observed between the second trimester NP group and the third trimester NP group (p = 0,003)

*2^a^ significant difference was observed between the second and third trimester NP group (p = 0,00), between the second trimester NP and the third trimester PPGP group (p = 0,00), between the second trimester PPGP and third trimester NP group (p = 0,00) as well as between the second trimester PPGP and third trimester PPGP group (p = 0,00), using the Post-Hoc Fischer LSD test

*^3^a significant difference was observed between the second trimester NP and third trimester PPGP group (p = 0,009) and between the third trimester NP and third trimester PPGP group (p = 0,02) using the Fischer LDS Post-Hoc test.

### Measurement of static postural stability

A Kistler 9286AA force plate and BioWare software were used. The force plate was equipped with a non-slip surface. An Airex™ (50 × 41 × 6 cm) foam pad was used for the compliant surface. Data were captured at a frequency of 200Hz and further analysed using Stab.Dat software [24]. Four variables were included: velocity of CoP excursion, CoP excursion in the mediolateral (ML) direction expressed as ML path length, CoP excursion in the anteroposterior (AP) direction expressed as AP path length and sway area calculated by principal component analysis (PCA) [25].

Participants were asked to step on the force plate and place their feet close together with their arms relaxed at their sides, to look at a point drawn on the wall in front of them, that was 2.5 meters form the testing area, and to remain still. Measurements were conducted in four sensory conditions. Each measurement followed the same order of sensory conditions: on a firm surface with eyes open and with eyes closed and on a compliant surface with eyes open and eyes closed. No practice trials were provided to the participants. Each measurement lasted up to 60 seconds or up to notable balance problems when participants moved arms or legs from the starting position. Between each trial a rest period of 30 seconds was given to the participants. Up to three attempts were conducted in each of the stated sensory conditions, and the best time result was considered for further analysis. If participants managed to maintain the standing position without visual balance problems for 60 seconds, measurement in that sensory condition was finished and the result was considered for further analysis. Measurement continued in the next sensory condition.

All participants managed to stand on the force plate for 60 seconds in all four sensory conditions. Therefore, 60 seconds of time series were further analysed.

### Statistical analysis

All data were analysed using the IBM SPSS 26 (Chicago, Illinois, USA) software. The Shapiro-Wilk test was used to determine if variables were normally distributed. Data showed normal distribution, so parametric tests were used for further analysis. Descriptive statistics included mean and standard deviation. To analyse the effect of gestation trimester and PPGP for all four postural sway variables, a two-way ANOVA for each experimental condition (standing on a firm surface with eyes open and closed and on a compliant surface with eyes open) was calculated followed by one way ANOVA with a post hoc Fischer LSD test to determine differences between pairs of groups. Postural sway variables were compared between four groups. Sample size and power calculation were not performed.

Pain intensity between the two PPGP groups was compared using a t-test for independent samples. Differences were considered significant when p<0.05. The correlation between number of falls and presence of PPGP variables was calculated using the Pearson correlation coefficient.

## Results

Demographic characteristics between the groups were similar except for number of births (p = 0.03), current week of gestation (p < 0.001) and frequent lifting (p = 0.04). The detailed results are presented in Table 1.

Thirteen pregnant women experienced a fall during pregnancy, a rate of 21%; one pregnant woman fell twice. The most common cause of the fall was a slippery surface (n = 7); three pregnant women fell while walking on stairs, three fell while carrying an additional load, and one fell while cycling. Ten pregnant women with PPGP, 5 in the second trimester and 5 in the third trimester of pregnancy, experienced a fall. A weak positive correlation was observed between presence of PPGP and the number of falls (r = 0.289; p = 0.02). Between the two groups with PPGP, no difference in pain intensity was observed (p = 0.86)

### Postural sway assessment

#### Firm surface eyes open

A two-way ANOVA revealed that there was no statistically significant interaction between the effects of gestation trimester and PPGP for all four postural sway variables (mean velocity (F1 = 1.317, p = 0.256), ML path length (F1 = 0.031, p = 0.861), AP path length (F1 = 0.197, p = 0.659), and sway area (F1 = 0.038, p = 0.846). Simple main effects analysis showed that the gestation trimester had a statistically significant effect on three postural sway variables (ML path length (p = 0.020), AP path length (p = 0.033), and sway area as calculated by PCA (p = 0.019)), while mean velocity did not have a statistically significant effect (p = 0.091). Simple main effects analysis showed that PPGP had a statistically significant effect on three postural sway variables (ML path length (p = 0.010), AP path length (p = 0.015) and sway area as calculated by PCA (p = 0.003)), while mean velocity did not have a statistically significant effect (p = 0.256).

#### Firm surface eyes closed

A two-way ANOVA revealed that there was no statistically significant interaction between the effects of gestation trimester and PPGP for all four postural sway variables (mean velocity (F1 = 0.051, p = 0.822), ML path length (F1 = 0.023, p = 0.879), AP path length (F1 = 0.009, p = 0.926), and sway area (F1 = 0.012, p = 0.913). Simple main effects analysis showed that gestation trimester had a statistically significant effect on all four postural sway variables (mean velocity (p = 0.018), ML path length (p = 0.009), AP path length (p = 0.015), and sway area as calculated by PCA (p = 0.005)). Simple main effects analysis showed that PPGP had a statistically significant effect on all four postural sway variables (mean velocity (p = 0.001), ML path length (p = 0.017), AP path length (p = 0.007), and sway area as calculated by PCA (p = 0.022)).

#### Compliant surface eyes open

A two-way ANOVA revealed that there was no statistically significant interaction between the effects of gestation trimester and PPGP for all four postural sway variables (mean velocity (F1 = 0.204, p = 0.654), ML path length (F1 = 0.174, p = 0.678), AP path length (F1 = 0.031, p = 0.861), and sway area (F1 = 0.336, p = 0.564). Simple main effects analysis showed that the gestation trimester had a statistically significant effect on two out of four postural sway variables (mean velocity (p = 0.002), ML path length (p = 0.031)), while AP path length (p = 0.084) and sway area as calculated by PCA (p = 0.276)) were not significant. Simple main effects analysis showed that PPGP had a statistically significant effect on all four postural sway variables (mean velocity (p = 0.035), ML path length (p = 0.021), AP path length (p = 0.016), and sway area as calculated by PCA (p = 0.008)).

#### Compliant surface eyes closed

A two-way ANOVA revealed that there was no statistically significant interaction between the effects of gestation trimester and PPGP for all four postural sway variables (mean velocity (F1< 0.001, p = 0.992), ML path length (F1 = 0.572, p = 0.452), AP path length (F1 = 0.312, p = 0.579), and sway area (F1 = 0.299, p = 0.587). Simple main effects analysis showed that the gestation trimester had a statistically significant effect on all four postural sway variables (mean velocity (p = 0.013), ML path length (p = 0.012), AP path length (p = 0.016), and sway area as calculated by PCA (p < 0.001)). Simple main effects analysis showed that PPGP had a statistically significant effect on all four postural sway variables (mean velocity (p = 0.007), ML path length (p = 0.018), AP path length (p = 0.013) and sway area as calculated by PCA (p = 0.015)).

To analyse the effect of PPGP for all four postural sway variables, a one-way ANOVA for each experimental condition was calculated, and postural sway variables were compared between four groups with post hoc tests.

#### Effect of PPGP

**Fig 1:**
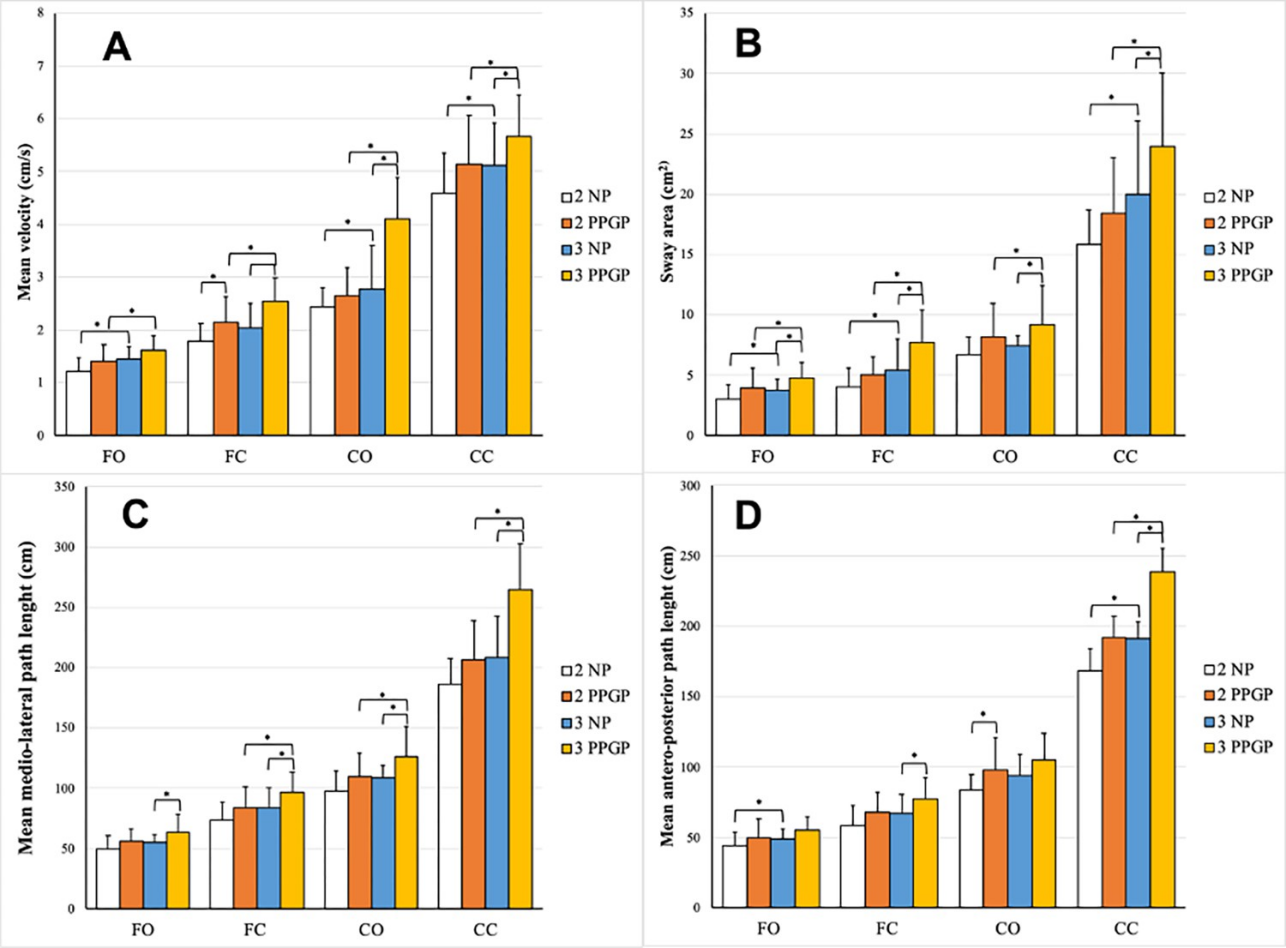
The comparison of mean velocity (A), sway area (B), medio-lateral path length (C) and antero-posterior path length (D) for the effect of trimester of pregnancy and for the effect of PPGP. FO–standing on firm surface with eyes open; FC–standing on firm surface with eyes closed; CO–standing on compliant surface with eyes open; CC–standing on compliant surface with eyes closed; 2NP–second trimester, no-pain group (white); 2PPGP–second-trimester pregnancy-related pelvic girdle pain group (orange); 3NP–third-trimester no-pain group (blue); 3PPGP–third-trimester pregnancy-related pelvic girdle pain group (yellow); * indicates a significant difference between the two groups p<0,05.

In the second trimester, significant differences in postural sway variables were observed between the two groups, with and without PPGP, for CoP velocity on a firm surface with eyes closed (p = 0.03), CoP path length in the AP direction on a compliant surface with eyes open (p = 0.04) (Fig 1).

In the third trimester, CoP velocity was significantly higher in the third trimester PPGP group in all conditions (p < 0.03) except for the firm surface, eyes-open condition (p = 0.08). The CoP path length in the AP direction was significantly higher in the third trimester PPGP group in both eyes closed conditions (firm surface: p = 0.04; compliant surface: p = 0.01). CoP path length in the ML direction was significantly higher in the third trimester PPGP group in all conditions (p < 0.03). Sway PCA area was significantly greater in the third trimester PPGP group in all sensory conditions (p < 0.05) (Fig 1).

#### Effect of advancement of pregnancy

Between pregnant women in the second and third trimester no–pain groups, statistically significant higher CoP velocity was observed in firm surface eyes open condition (p = 0.02) and both compliant surface conditions (eyes open: p = 0.00; eyes closed p = 0.02). CoP velocity was significantly higher in the third-trimester group with PPGP, compared to the second-trimester PPGP group in all sensory conditions (p < 0. 04). No difference was observed in CoP path length in the ML direction between both no–pain groups (p > 0,08). CoP path length in the ML direction was significantly greater in the third-trimester PPGP group compared to the second trimester PPGP group in all sensory conditions (p < 0.04) except for the firm surface eyes open condition (p = 0.7). Significant differences in the CoP AP path length between both groups with no pain were observed in the firm surface, eyes open (p = 0.03) and compliant surface, eyes-closed condition (p = 0.01). No difference was observed in the CoP AP path length between both groups with PPGP (p > 0.07), except for the compliant surface, eyes-closed condition (p = 0.007). A larger sway area was observed in the third-trimester no-pain group compared to the second trimester no-pain group in both firm surface conditions (eyes open: p = 0.02; eyes closed: p = 0.01) and in the compliant surface, eyes-closed condition (p = 0.03). Between both PPGP groups larger sway area was observed all sensory conditions (p < 0.005) (Fig 1).

## Discussion

This study indicates that pregnant women with PPGP demonstrated poorer static stability compared to pregnant women without PPGP, especially in the third trimester of pregnancy. Our results indicate that pregnant women with PPGP have poorer static stability, similar to pregnant women with low back pain based on reports of Öztürk and colleagues [26], who concluded that pregnant women with low back pain have poorer static stability compared to pregnant women without pain in the third trimester of pregnancy. Öztürk and colleagues [26] excluded pregnant women with PPGP based on testing and differential diagnosis proposed in the European guidelines for the diagnosis of pelvic girdle pain [16].

In our study pregnant women with PPGP in the third trimester of pregnancy had a statistically significant poorer static stability when compared to the third trimester no-pain group, while the differences between the two-second trimester groups were not as conclusive. Pregnant women with PPGP have an asymmetric laxity of the sacroiliac joints [27] and a lower ability to stabilise the pelvic girdle [28, 29]. Greater CoP excursion and poorer balance can be explained by the lower stabilisation of the pelvic girdle in pregnant women with PPGP. Pelvic girdle pain affects the pelvic stabilisation function, disturbances in the automatic movement patterns are present, changes can be seen in the posture and gait pattern [30], which can directly affect postural stability.

Our results showed that pregnant women in the third trimester of pregnancy with PPGP had significantly greater CoP excursion in the ML direction when compared to the third trimester no-pain group and the second trimester PPGP group. Greater CoP excursion is a consequence of the delayed muscle activation as a response to balance problems observed in people with pelvic girdle pain [31]. Impaired function of the proprioceptive system and difficulty perceiving body position was observed in adult women with pelvic girdle pain, who also had movement coordination and balance issues [32].

In eyes-closed conditions, greater CoP excursion was observed in pregnant women with PPGP. Pregnant women tend to rely more on visual cues than on cues from the proprioceptive system [9]. People with pelvic girdle pain rely more on visual cues for movement planning and trunk stabilisation [33]. Muscle onset delay in stabilising muscles and abnormal anticipatory postural adaptations were observed [31, 33].

Based on our results, no significant differences in postural stability were observed between both groups in the second trimester of pregnancy. In the second trimester of pregnancy, musculoskeletal changes, that normally occur during pregnancy advancement, are not as prominent as in the third trimester of pregnancy [2, 5]. Levels of the hormone relaxin are not as high in the second as in the third trimester of pregnancy [34]. Higher levels of the hormone relaxin contribute to the laxity of the pelvis and reduce the ability to stabilize the pelvis [35]. Non conclusive results from our study, could also be explained by the nature of PPGP that normally occurs between the 17^th^ and the 19^th^ week of gestation [20]. It could be possible, that the effect of PPGP on balance and the ability to stabilize the trunk and pelvis was not yet pronounced enough to be noticeable during measurements in the second trimester.

In our study pregnancy advancement significantly impacted postural control regardless of PPGP. In previously published studies, authors concluded that pregnant women in their third trimester of pregnancy have greater sway area, velocity of CoP excursion and difficulty maintaining a stable stance position compared to women in the second trimester of pregnancy [9–11, 33]. Due to the centre of mass position change, which is most dominant in the third trimester of pregnancy, body mass distribution is altered [36, 37]. Ligament laxity also increases with the advancement of pregnancy, and changes in the proprioceptive system occur as a consequence [37].

The majority of pregnant women do not receive any information on preventing falls during pregnancy, even though the information would be appreciated [38]. Pregnant women should be educated and advised on musculoskeletal changes, risk factors for falls [8] and possible strategies to prevent falls during the early stages of pregnancy and within each trimester [39]. Physical activity is a possible fall prevention strategy in pregnancy. Regular physical activity helps women learn and adapt to the changes their body experiences during pregnancy. They can form stabilisation strategies to maintain a stable position in different balance disorders. In other studies, physical activity was also proposed as a possible fall prevention intervention [11, 26, 40–42].

One of the limitations of the study was the small sample size. We compared two different groups of women, one in the second and one in the third trimester of pregnancy, instead of performing a longitudinal study in the group of pregnant women at different trimesters. Another limitation is the small difference in gestation weeks between the second and third trimester groups, which was due to the nature of PPGP that usually appears at the end of the second trimester. In our study only static stability was measured and observed. No practice trials were allowed; therefore, participants were not able to form stabilization strategies before measurement begun, which could be another limitation of our study.

Further studies are needed to assess the effect of PPGP on postural stability during pregnancy. Large, longitudinal, high-quality studies are needed to further asses if PPGP affects static postural stability. Future studies should also focus on the dynamic balance in pregnant women with PPGP. The effect of a pelvic belt and pelvic stabilisation exercises, that are the usual course of treatment in pregnant women with PPGP, on static and dynamic postural stability in pregnant women with PPGP should also be examined.

## Conclusion

Pregnant women with PPGP demonstrated poorer static stability compared to pregnant women without PPGP, especially in the third trimester of pregnancy. This study suggests that women with PPGP may be at a higher risk of falling. Pregnant women with PPGP relied more on visual cues to maintain a stable position. PPGP affected especially the mean velocity of CoP excursion. With the advancement of pregnancy, static stability decreased, regardless of PPGP. Pregnant women with PPGP fell more often than pregnant women without PPGP and had poorer static stability, so information on balance issues and fall prevention interventions should be included in the regular treatment of pregnant women with PPGP.

## Supporting information

S1 File Postural sway assessment. FO–standing on firm surface, eyes open; FC–standing on firm surface, eyes closed; CO–standing on complian surface, eyes open; CC–standing on compliant surface, eyes closed; NP–no pain; PPGP–pregnancy related pelvic girdle pain; SD–standard deviation; p–p value; p 2NP– 3NP–p value between second trimester no pain group and third trimester no pain group; ML–medio lateral; AP–antero posterior; * indicates a significant difference.
(PDF)
