# Supplementary material for: Pregnancy-related pelvic girdle pain affects balance in the second and third trimesters of pregnancy
Source: PLoS One. 2024 Mar 8;19(3):e0287221. doi: 10.1371/journal.pone.0287221 (PMC10923470; doi:10.1371/journal.pone.0287221)
Supplement: S1 File — FO–standing on firm surface, eyes open; FC–standing on firm surface, eyes closed; CO–standing on complian surface, eyes open; CC–standing on compliant surface, eyes closed; NP–no pain; PPGP–pregnancy related pelvic girdle pain; SD–standard deviation; p–p value; p 2NP– 3NP–p value between second trimester no pain group and third trimester no pain group; ML–medio lateral; AP–antero posterior; * indicates a significant difference. (PDF) [file pone.0287221.s001.pdf]

Condition	Group		Velocity (cm/s)	Excursion ML (cm)	Excursion AP (cm)	PCA (cm^2)
FO	2nd trimester	NP (mean ± SD)	1,21 ± 0,26	49,19 ± 11,59	44,16 ± 9,71	3,05 ± 1,17
		PPGP (mean ± SD)	1,41 ± 0,31	56,16 ± 9,83	49,99 ± 12,84	3,97 ± 1,58
		p	0,06	0,09	0,11	0,05*
	3rd trimester	NP (mean ± SD)	1,44 ± 0,24	55,37 ± 6,45	49,21 ± 6,58	3,75 ± 0,96
		PPGP (mean ± SD)	1,61 ± 0,28	63,34 ±14,59	55,39 ± 9,43	4,79 ± 1,28
		p	0,08	0,04*	0,08	0,02*
	p 2NP – 3NP		0,02*	0,13	0,03*	0,02*
	p 2PPGP – 3PPGP		0,04*	0,73	0,13	0,05*
FC	2nd trimester	NP (mean ± SD)	1,78 ± 0,35	73,56 ± 14,51	58,63 ± 13,83	4,04 ± 1,58
		PPGP (mean ± SD)	2,14 ± 0,49	84,01 ± 17,09	68,25 ± 13,74	5,05 ± 1,47
		p	0,03*	0,08	0,07	0,21
	3rd trimester	NP (mean ± SD)	2,03 ± 0,46	83,97 ± 16,21	67,18 ± 13,31	5,39 ± 2,57
		PPGP (mean ± SD)	2,55 ± 0,43	97,07 ±15,97	77,45 ±15,09	7,69 ± 2,69
		p	0,01*	0,02*	0,04*	0,01*
	p 2NP – 3NP		0,12	0,08	0,09	0,01*
	p 2PPGP – 3PPGP		0,009*	0,02*	0,07	0,007*
CO	2nd trimester	NP (mean ± SD)	2,43 ± 0,37	97,89 ± 16,25	83,52 ± 11,58	6,68 ± 1,49
		PPGP (mean ± SD)	2,64 ± 0,53	109,19 ± 20,06	97,52 ± 23,69	8,16 ± 2,79
		p	0,4	0,18	0,04*	0,08
	3rd trimester	NP (mean ± SD)	2,78 ± 0,82	108,28 ± 10,89	94,29 ± 14,94	7,47 ± 0,82
		PPGP (mean ± SD)	4,11 ± 0,77	126,19 ± 24,51	104,69 ± 19,49	9,21 ± 3,16
		p	0,01*	0,03*	0,11	0,03*
	p 2NP – 3NP		0,00*	0,21	0,10	0,25
	p 2PPGP – 3PPGP		0,00*	0,04*	0,27	0,01*
CC	2nd trimester	NP (mean ± SD)	4,59 ± 0,76	186,11 ± 20,81	168,63 ± 15,45	15,86 ± 2,87
		PPGP (mean ± SD)	5,14 ± 0,92	206,46 ± 32,04	192,09 ± 14,79	18,40 ± 4,59
		p	0,06	0,28	0,18	0,18
	3rd trimester	NP (mean ± SD)	5,11 ± 0,81	208 ± 34,45	191,24 ± 11,91	20,01 ± 6,06
		PPGP (mean ± SD)	5,66 ± 0,78	264,57±38,18	238,94 ± 16,36	23,95 ± 6,04
		p	0,03*	0,01*	0,01*	0,03*
	p 2NP – 3NP		0,02*	0,23	0,01*	0,02*
	p 2PPGP – 3PPGP		0,01*	0,002*	0,007*	0,007*
